# Effect of Biopolymer Additives on Functional Properties of Alginate-Based Composite Hydrogels

**DOI:** 10.3390/gels12030266

**Published:** 2026-03-22

**Authors:** Tanja Krunic, Nevena Ilic, Andrea Osmokrovic

**Affiliations:** 1 Innovation Center of the Faculty of Technology and Metallurgy Ltd., University of Belgrade, Karnegijeva 4, 11000 Belgrade, Serbia; 2 Faculty of Technology and Metallurgy, University of Belgrade, Karnegijeva 4, 11000 Belgrade, Serbia

**Keywords:** whey protein, gelatin, pectin, starch, chitosan

## Abstract

Hydrogels constructed from natural biomacromolecules with multifunctional properties, such as improved mechanical strength, ionic stability, biocompatibility, and ionic conductivity, are highly desirable for advanced food and biomedical applications, yet remain challenging to design. Although alginate is one of the most widely used hydrogel-forming polysaccharides due to its biocompatibility and gelation ability, its intrinsic limitations often hinder the development of hydrogels with fully optimized performance. This review provides a systematic comparison of alginate-based composite hydrogels formed with complementary biopolymers, including whey proteins, gelatin, pectin, starch, and chitosan, focusing on their synergistic effects on structural, mechanical, and functional properties. Recent studies are critically analyzed to elucidate how polymer–polymer interactions influence gel network formation, environmental ionic stability, and encapsulation performance. Particular attention is given to fabrication strategies and formulation parameters that enhance the immobilization and controlled release of probiotics, vitamins, polyphenols, and other bioactive compounds. By integrating current knowledge on structure–function relationships and processing approaches, this review offers practical design guidelines for the development of multifunctional alginate-based hydrogel systems for applications in functional foods and nutraceutical delivery.

## 1. Introduction

In recent decades, a surge of research on “smart” hydrogels that respond to various stimuli has led to many novel applications of these materials. Hydrogels are composed of hydrophilic polymers that are cross-linked into an insoluble but highly water-absorbent structure [1,2]. Accordingly, hydrogels have emerged as a highly versatile class of materials with applications across diverse fields, including biomedical engineering (tissue engineering, drug delivery, wound dressings), food science (encapsulation, texture modification, carrier systems), and environmental engineering (adsorbents, sensors), among others [2,3,4,5]. The cross-links forming the hydrogel network can be strong chemical bonds or weak physical ones [1,2].

Hydrogels can be classified according to several criteria, including their origin (natural or synthetic), cross-linking mechanism (physical/ionic or chemical/covalent), degradability (permanent or biodegradable), and responsiveness (stimuli-responsive or “smart” hydrogels). Natural hydrogels, derived from biopolymers such as alginate, gelatin, and chitosan, exhibit inherent biocompatibility and biodegradability; however, they often display limited mechanical strength and batch-to-batch variability [6].

Among the various fabrication techniques for hydrogel particles, extrusion, also referred to as extrusion-dripping or extrusion-gelation, is one of the most widely employed due to its simplicity, scalability, and mild processing conditions. In this method, a polymer solution is extruded through a nozzle, syringe, or pipette into a gelling bath containing cross-linking ions. The resulting droplets undergo instantaneous or gradual gelation to form spherical or near-spherical hydrogel particles [4]. The morphology, size, and uniformity of the produced particles are influenced by parameters such as nozzle diameter, extrusion flow rate, droplet spacing, cross-linker concentration, and gelling bath composition. This technique is particularly advantageous when precise control of particle size, gentle processing conditions (especially for encapsulated biomolecules or cells), and operational simplicity are desired. Furthermore, extrusion-based methods are highly compatible with applications in the food, pharmaceutical, and biomedical fields [7].

**Figure 1:**
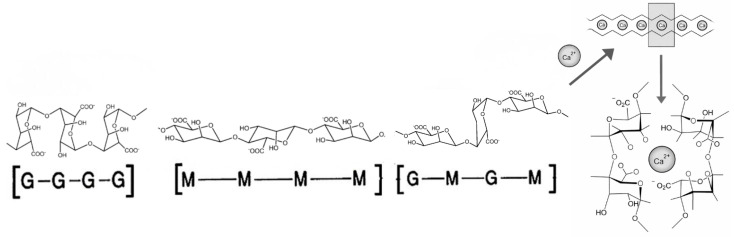
Three types of M-G combination in the alginate molecule and “egg-box” formation with calcium ions.

Among naturally occurring hydrogel-forming polymers, sodium alginate and its ionically cross-linked derivative, calcium alginate, hold a prominent position. Alginates with a wide range of compositions can be obtained from bacteria such as *Azotobacter vinelandii* and *Pseudomonas* spp., or commercially from brown marine macroalgae such as *Laminaria* sp., *Durvillaea* sp., and *Sargassum* sp. [8]. Alginate is a linear copolymer consisting of β-D-mannuronic (M) and α-L-guluronic (G) acid residues arranged in homopolymeric and heteropolymeric block sequences (Figure 1).

The source of alginate determines the G-to-M ratio and the molecular weight of the polysaccharide, resulting in significant differences in the physicochemical and mechanical properties of the gels. In the presence of divalent cations, particularly calcium ions, the G-block regions interact to form the characteristic “egg-box” junction zones that promote gelation and establish the hydrogel network. Alginate affinity toward polyvalent cations is directly dependent on the number of G-blocks present in its structure [9]. Typically, the stability and gel strength of hydrogels increase with a higher content of α-L-guluronic acid in the alginate molecules. Alginate gelation processes with H^+^ or Ca^2+^ are the most common in food applications. The H^+^-type alginate gels can be formed when the pH of alginate solutions is below the pKa of the uronic acid residues [10]. The calcium ion gelation can be performed in a wide pH range, obtaining different properties. In particular, the calcium alginate gel obtained at pH 3.8 has stronger chain interactions and hence a denser and more interconnected microstructure than calcium alginate gels obtained at a pH higher than 5.0 [11].

Alginate has been selected as the central hydrogel matrix in this review because it represents one of the most widely used and well-established polysaccharide hydrogels in food-related encapsulation systems. Its popularity arises from several advantageous characteristics, including biocompatibility, low toxicity, food-grade status, mild and controllable gelation conditions, and the ability to form hydrogels through simple ionic cross-linking with divalent cations such as Ca^2+^. These properties enable the encapsulation of sensitive bioactive compounds without exposure to harsh processing conditions and allow for the formation of hydrogel carriers with tunable structural properties. Consequently, alginate has become one of the most frequently used matrices for the immobilization and delivery of probiotics, vitamins, polyphenols, proteins, and other bioactive compounds in food systems [12]. Despite these advantages, alginate hydrogels also exhibit several inherent limitations, such as relatively high porosity, limited mechanical stability, sensitivity to acidic environments, and insufficient control over the release of encapsulated molecules [4,13]. The alginate gels exhibit low resistance to acidic environments and tend to shrink, resulting in syneresis and the subsequent expulsion of water-soluble compounds from within the gel network. Syneresis may also occur during gel formation as a consequence of electrostatic interactions between calcium ions and the negatively charged carboxylate groups of alginate. For instance, gelation of 1% sodium alginate has been reported to cause a 44% loss of water and a 36% loss of encapsulated lactase enzyme [13]. Importantly, many of these drawbacks can be effectively mitigated by combining alginate with other biopolymers that introduce additional intermolecular interactions and structural reinforcement within the hydrogel network. For this reason, composite alginate hydrogels have been widely investigated as a strategy to improve carrier stability, reduce permeability, and tailor release behavior. Being food-grade and widely accepted in food applications, alginate has been extensively used for encapsulating proteins, antioxidants, polyphenols, vitamins and probiotics usually in combination with polymers with similar characteristics that can improve alginate hydrogel carriers [4,7,14,15].

Accordingly, this review focuses on the biopolymers most commonly used as additives to alginate matrices, namely whey protein, gelatin, starch, pectin, and chitosan, which represent some of the most frequently reported food-grade polymers capable of modifying alginate hydrogel structure and functionality. By summarizing their interaction mechanisms with alginate and the resulting effects on hydrogel properties, this review aims to highlight how specific polymer combinations can be used to design improved hydrogel carriers for food-related encapsulation applications.

## 2. Biopolymer Additives for Alginate-Based Composite Hydrogels

### 2.1. Whey Protein

**Figure 2:**
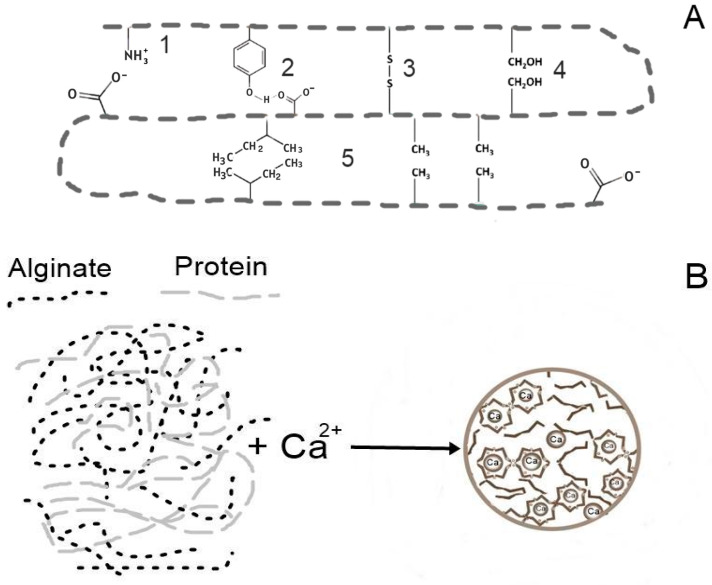
(**A**) A schematic representation of stabilizing interactions in a protein: (1) electrostatic interactions, (2) hydrogen bonds, (3) disulfide bonds, (4) dipole–dipole interactions, and (5) hydrophobic interactions; (**B**) alginate–protein hydrogel.

Whey protein is a mixture of globular proteins isolated from whey as a byproduct of cheese production. Whey protein has calcium-binding sites that can bind with calcium ions. Moreover, α-lactoalbumin has especially strong calcium-binding sites [16]. The ability of whey proteins to form gels and microcapsules without the use of severe heat treatment or any chemicals makes them an attractive material for encapsulation in the food industry, especially as an effective carrier for the protection of probiotic organisms [14,17]. The functional properties of whey proteins, particularly their capacity for thermo-reversible gelation and their high affinity for divalent cations, enable their application as structural components in processes such as extrusion technology. The stabilizing interactions in a protein are electrostatic interactions, hydrogen bonds, disulfide bonds, dipole–dipole interactions, and hydrophobic interactions (Figure 2), but protein or peptide aggregation usually occurs in three different ways: electrostatic shielding, ion/hydrophobic interactions, and cross-linking with negatively charged carboxylic groups of whey protein molecules via protein–cation–protein bridges [18]. However, the specific technological and chemical properties of whey proteins exhibit polymorphism, varying significantly based on source material (e.g., sweet vs. acid whey), isolation methodology (e.g., microfiltration, ion-exchange chromatography), and processing history (e.g., thermal denaturation, pH adjustments). Whey protein alone, without the addition of any agents, builds mechanically insufficiently strong and suboptimally stable matrices. Whey proteins and their peptides, which share comparable functional attributes, demonstrate a similar mechanism of action in matrix formation [14]. Controlled enzymatic hydrolysis is a highly effective methodology for improving the techno-functional profile of the proteins. This modification leads to favorable alterations in key parameters, including enhanced solubility, increased surface hydrophobicity, improved fat/oil binding capacity, and modified zeta-potential [14,19]. These controlled improvements are essential for engineering whey protein and peptide carriers with superior performance and tailored delivery characteristics [14,15].

Whey protein isolate (WPI) and concentrate (WPC) are used in various functional food applications due to their emulsification, gelation, thickening, foaming and water-binding properties, and excellent nutritional value, as well as the ability to carry hydrophobic substances [17,19]. A very important property of whey protein, which makes it an often-used carrier for oil materials, is emulsification. Due to their amphiphilic character, whey proteins are able to behave as surface-active agents. As a result, they quickly adsorb at the oil–water interface of emulsions, where they associate with one another and generate a continuous and uniform interfacial film surrounding the oil droplets, primarily stabilized by intermolecular β-sheet interactions. Then, gelation of these structures can be promoted by both calcium ions and heat treatment [20,21]. Whey protein and alginate interact to form complexes and hydrogels through electrostatic interactions (dominant when the polymers have opposite charges, depending on pH), hydrogen bonding, and hydrophobic interactions [19,21]. Emulsion-gelled microparticles are a class of soft solid particles of small size. These particles are characterized by emulsion droplets entrapped inside a soft semi-solid matrix, which provides control of the mechanical properties and delivery mechanisms [22]. Zhao et al. [23] developed a superior delivery system by constructing a WPC-based emulsion gel synergistically modified with alginate and ultrasound. Alginate incorporation in whey protein improves a primary gel network through hydrophobic interactions, hydrogen bonding, and disulfide bridging, significantly improving gel hardness, water-holding capacity, and rheological properties. Whey protein and alginate create a denser gel network, which enhances physical stability and provides superior protection of astaxanthin during digestion [23]. Espinaco et al. [24] examined the difference between alginate and alginate–WPC particles used for chia oil and astaxanthin encapsulation. Both types of particles protected the bioactive compounds during the gastric phase, releasing less than 10% in this phase and allowed up to 90% release during the intestinal phase. In that study, the type of wall material did not influence the bioaccessibility of bioactive compounds. Pedrali et al. [21] reported that alginate and whey proteins exhibit strong interactions, leading to the formation of hydrogels whose properties can be tuned by factors such as alginate molecular weight, the M:G ratio, pH, and the presence of calcium ions or transglutaminase. Alginate–whey protein hydrogels, prepared as beads, microparticles, microcapsules, or nanocapsules, generally exhibit improved encapsulation efficiency and more favorable release behavior for antioxidants compared with hydrogels composed solely of alginate [21]. Wichchukit et al. [25] incorporated gel particles into a viscous matrix mimicking a food material for sustained delivery purposes. Rheological characterization showed that adding whey protein to alginate yielded pseudoplastic and viscoelastic behavior of the gel solution. Mechanical relaxation tests of the gel particles quantified the viscoelastic property; pure whey protein particles tended to be more solid and relaxed the least. Pure alginate particles did not have sufficient structural integrity. The combination of whey protein and alginate formed particles with good integrity for the release of riboflavin vitamin [25].

A combination of alginate and whey protein, used as a carrier for probiotics obtained by electrostatic extrusion, allows for better fermentative activity and probiotic viability during storage and SGI conditions compared to alginate [4,14]. The incorporation of WPC into the alginate hydrogel matrix leads to a significant enhancement in the mechanical properties of the carrier. Conversely, the use of whey protein hydrolysate (WPH) in place of WPC also improves the mechanical characteristics, though the resulting improvement is typically less pronounced compared to the WPC–alginate composite matrix [4,14]. This difference is caused by the reduced molecular size and structural complexity of the peptides in the hydrolysate, which may limit their ability to participate effectively in the extensive inter-chain cross-linking network formation. Although the mentioned enrichment of alginate carriers has a positive effect on the mechanical properties of the particles, when it comes to fermentative activity and carrier porosity, the additives have different effects. The addition of WPC and WPH has a positive effect on fermentation but also increases the release of probiotics from the matrix due to higher carrier porosity [4,14]. As can be seen in Table 1, the increase in mechanical properties of alginate-based hydrogel particles when WPC or WPH were added was even more pronounced after the fermentation process. The mechanical strength of the particles increased most in the WPC–alginate carrier, somewhat less in the WPH–alginate carrier, and least in particles containing only alginate as a probiotic carrier. The change in carrier properties due to different interactions of whey proteins/peptides and alginates was also shown through the difference in the porosity of hydrogel particles. The porosity of the hydrogel particles was evaluated indirectly through the release behavior of immobilized probiotics into the surrounding medium. Among the tested formulations, the WPC–alginate hydrogels exhibited the highest probiotic release, indicating greater porosity. In contrast, the WPH–alginate hydrogels showed moderately lower release and porosity, suggesting that hydrogel porosity is directly related to the molecular size of the incorporated protein polymer. Specifically, longer amino acid chains appear to promote the formation of a more porous network structure. The lowest porosity was observed in alginate hydrogels without the addition of whey proteins or peptides. The different interactions between alginate and whey proteins or peptides were confirmed by FTIR analysis [14,26].

Alginate particles with added whey proteins and peptides also showed different behavior during simulated gastrointestinal digestion. Particles with whey proteins and protein hydrolysate showed better protection of probiotics, but also different mechanical properties during the digestion process [14,27]. Remarkably, the WPC–alginate particles that exhibited a high probiotic release (≈19.5%) provided enhanced protection during gastrointestinal digestion compared to alginate particles with a considerably lower release (≈3.5%) [14].

FTIR analysis showed that the metabolic activity of the encapsulated cultures was differently affected by the carrier containing hydrolyzed and non-hydrolyzed whey protein [14,26]. Ten et al. [28] showed that the most dominant interactions between whey protein and alginate are hydrogen bonding and electrostatic attractions. SEM analysis confirmed the different structure and porosity of the particles with and without whey protein [27].

In general, whey protein is the most common protein used in combination with alginate as a carrier in the food industry. Whey protein with alginate makes a good carrier for oil-phase encapsulation, as well as polyphenols and antioxidants. Whey protein and alginate create a denser gel network, which enhances physical stability and provides superior protection of astaxanthin during digestion [23]. Hydrogels of alginate and whey proteins, in the forms of beads, microparticles, microcapsules, and nanocapsules, generally provide better encapsulation efficiency and release properties for antioxidants with respect to the hydrogel of alginate alone [21]. The addition of WPC and WPH into the alginate matrix improves hydrogel properties for probiotic encapsulation [14], and the incorporation of WPC and WPH into the alginate matrix used for probiotic encapsulation significantly enhances the antioxidant capacity of both the carrier and the food product [15]. The combination of whey protein and alginate formed particles with good integrity for the encapsulation and the release of vitamins and medicaments [25,28].

**Table 1:** Mechanical strength of the carrier used for probiotic encapsulation during whey-based substrate fermentation measured by compression testing of packed beads * [4,14] and single beads ** with a diameter of about 2.5 mm in coconut milk [29].

	Maximal Force, Before Fermentation, N	Maximal Force, After Fermentation, N	Cell Release During Fermentation, %	References
Alginate *	2.1741 ± 0.015	2.3841 ± 0.024	3.44 ± 0.19	[4]
Alginate + WPC *	2.3861 ± 0.041	2.9541 ± 0.057	19.70 ± 0.78	[14]
Alginate + WPH *	2.3591 ± 0.021	2.781 ± 0.030	6.98 ± 0.23	[14]
Alginate + chitosan *	2.873 ± 0.021	2.963 ± 0.027	2.48 ± 0.10	[4]
Alginate + pectin **	0.6221 ± 0.0505	0.2283 ± 0.0095	/	[29]
Alginate + pectin + flaxseed **	0.1883 ± 0.0158	0.2382 ± 0.0408	/	[29]
Alginate + flaxseed **	0.3451 ± 0.0504	0.2239 ± 0.0169	/	[29]

### 2.2. Gelatin

**Figure 3:**
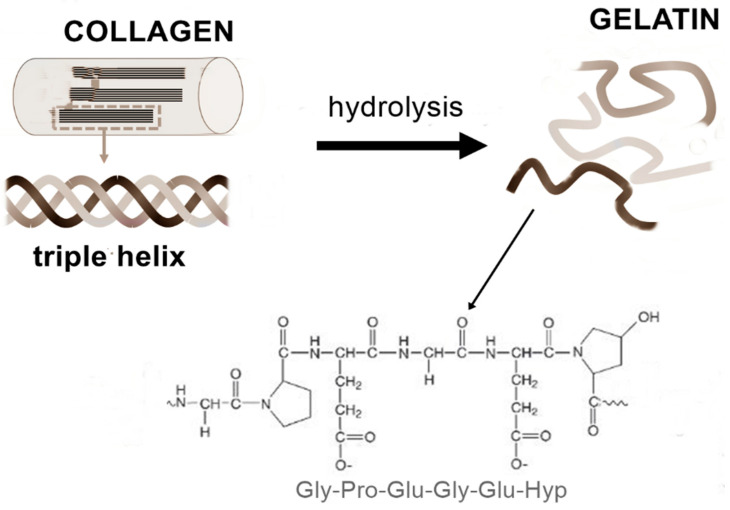
Gelatin produced by denaturation of collagen with a gelatin Gly–A–B structure.

Gelatin is a protein obtained by the partial hydrolysis and denaturation of the triple helix of collagen, maintaining a molecular composition that closely resembles that of the initial protein (Figure 3). Its structure is characterized by repeating amino acid triplets, Gly–A–B, where glycine is consistently present, and A and B are typically proline and hydroxyproline residues. This unique sequence arrangement is fundamental to the gelling ability of gelatin. The polypeptide chains contain reactive side groups such as carboxyl (–COOH) and amino (–NH_2_) functionalities, which can interact with acids, bases, aldehydes, metal ions, and surfactants. Cross-linking between protein chains can alter gelatin’s physicochemical characteristics, influencing mechanical properties such as tensile strength, elasticity, and degradation resistance, as well as permeability and interfacial interactions [30,31,32,33].

**Figure 4:**
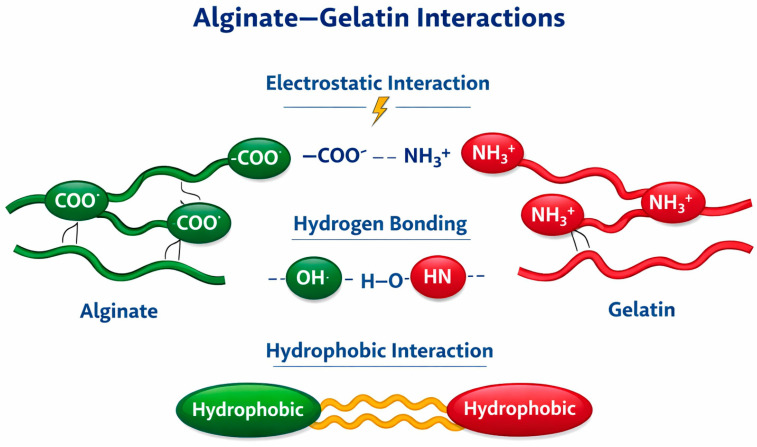
Illustration of alginate–gelatin interactions.

Because of the thermoreversible nature of gelatin gels and their limited mechanical stability, a common approach to enhance gelatin’s structural and functional performance involves blending it with other polymers. Gelatin is frequently combined with alginate to integrate their complementary properties synergistically. Gelatin contributes bioactivity through the presence of cell-binding, while alginate provides structural integrity and mechanical reinforcement [34,35]. Gelatin and alginate interact primarily through electrostatic interactions and hydrogen bonds. The interaction’s nature is dependent on factors like pH, temperature, ionic strength, and the specific biopolymer concentrations, which can result in different gel structures, such as phase-separated networks or coacervates [36]. Although both whey proteins and gelatin are proteins, their interactions with alginate differ. The interaction of alginate with whey proteins is more dependent on pH and micelle formation, involving a combination of electrostatic and hydrophobic interactions, whereas the interaction with gelatin is more predictable and stable, predominantly electrostatic and hydrogen-bond mediated, leading to the formation of firm, networked gels (Figure 4) [16,17,18,36].

Skopińska-Wiśniewska et al. [33] demonstrated that a multi-stage cross-linking approach markedly alters the structural and mechanical characteristics of gelatin–alginate hydrogels. The combined stabilization of alginate by calcium ions and the chemical cross-linking reactions involving gelatin resulted in the development of networks with modified architecture. The incorporation of gelatin, particularly when chemically cross-linked, was found to interfere with the typical formation of alginate “egg-box” structures induced by calcium ions. As a result of these concurrent interactions, the obtained hydrogels exhibited enhanced uniformity, more balanced cross-linking density, and improved flexibility [33].

Panouille and Larreta-Garde [36] investigated how mixtures of alginate and gelatin behave under different environmental conditions and identified the circumstances under which a mixed gel can form. They observed that alginate gels may collapse when exposed to elevated polymer or calcium levels, increased ionic strength, or temperatures between 35 and 45 °C. This collapse was attributed either to phase separation within the mixture or to excessive alginate association promoted by high calcium and total biopolymer concentrations. Under other sets of conditions, the two polymers were able to form a combined network, in which the alginate gel remained thermally irreversible while the gelatin component preserved its reversible, thermosensitive behavior.

Goudoulas and Germann [37] provided comprehensive rheological data for binary aqueous gelatin–alginate systems with concentration ratios approaching 1:1. At 5 °C, all mixed gels displayed a higher storage modulus than either of the single-polymer gels. Their findings indicate that formulations containing up to 5% gelatin and 2.5% alginate produced uniform gels, whereas the system composed of 5% gelatin and 5% alginate likely approached the phase separation regime. The influence of polysaccharide concentration on the sol–gel and gel–sol transitions has been examined for systems containing up to 5% alginate. Under small-amplitude oscillatory shear conditions, both transitions were observed to shift toward higher temperatures with increasing polysaccharide content, indicating enhanced thermal stability of the gel network. Kinetic measurements further showed that the storage modulus (G′) continued to increase over time under isothermal conditions, although the rate of increase gradually diminished as the network approached equilibrium. Moreover, binary gels consistently exhibited higher storage modulus values compared to pure gelatin gels at 5 °C, with increases of approximately 9% and 24% for systems containing 5% and 2.5% gelatin, respectively, suggesting that polysaccharide incorporation contributes to additional network reinforcement.

The incorporation of alginate was found to modify the intrinsic hydrophobic interactions within gelatin [38]. A reduction in surface hydrophobicity, together with an increase in fluorescence intensity in gelatin–alginate mixtures, indicates that hydrophobic amino acid residues become less exposed as aggregates form in the presence of alginate. Because β-strand assembly in unfolded polypeptides is often promoted by hydrophobic interactions among adjacent side chains [39], these observations suggest that the gelatin–alginate polyelectrolyte complex is formed mainly through hydrogen bonding, with accompanying rearrangements in hydrophobic domain interactions.

In their work, Lee et al. [38] identified a gelatin: alginate ratio of 1.9 as optimal for producing a stable emulsion gel. However, the ideal ratio varies across studies. For example, Wang et al. [40] reported an optimum of 3.33, while Derkach et al. [41,42] found ratios of 14.3 for fish gelatin and 1.05 for bovine gelatin for the most effective interaction with alginate. Such discrepancies likely arise from differences in the formation and stability of gelatin–alginate polyelectrolyte complexes, which are influenced by factors including polymer charge distribution, chain flexibility, molecular mass, ionic strength, pH, and temperature [41].

In addition to the components used to form the hydrogel, the rheological properties and gelling ability are also influenced by the compounds being encapsulated. According to Thakur et al. [43], the mechanical characteristics of gelatin–alginate hydrogels used for antioxidant encapsulation may be improved by the interaction between gelatin, alginate, and polyphenols of plant extracts. Polyphenols present in natural extracts are capable of interacting with the components of the polymer matrix through both covalent and non-covalent interactions [44].

Chaari et al. [45] developed antioxidant bioactive films composed of gelatin–alginate matrices incorporating aqueous beetroot peel extract (BPE). The influence of adding BPE at concentrations of 0.25, 0.5, and 1% on the films’ mechanical, physical, antioxidant, and antibacterial properties was investigated. An increase in BPE concentration resulted in improved mechanical and physical characteristics of the films. The swelling of gelatin–alginate hydrogel was high (88%), and the addition of BPE decreased it. This observation is consistent with the findings of Aydin [46], who reported that higher concentrations of *Hibiscus sabdariffa* extract led to a reduction in swelling capacity. This effect may be attributed to interactions between plant-derived polyphenols and the carboxyl groups of alginate, which likely reduced the availability of these groups for interaction with water molecules [46]. In comparison, the mechanical properties of the gelatin–alginate film were initially high and further increased by adding BPE.

A review of the literature has shown that the alginate–gelatin system is predominantly used for encapsulating and protecting bioactive compounds, antioxidants and polyphenols, due to the synergistic combination of alginate’s ionic gelation and gelatin’s protein-based network. This hybrid matrix is frequently used to improve gastrointestinal stability, provide controlled release, and enhance the oxidative and thermal protection of sensitive compounds and probiotics [38,47,48]. In addition, alginate–gelatin blends are widely used for edible films and coatings, where gelatin improves flexibility and mechanical behavior, while alginate contributes to barrier and structural stability [43,46,47]. These biopolymers are also employed in hydrogel emulsion systems [38,49], structure design for functional foods, and emerging food 3D-printing applications due to their tunable rheological and gelling properties [49].

### 2.3. Starch

**Figure 5:**
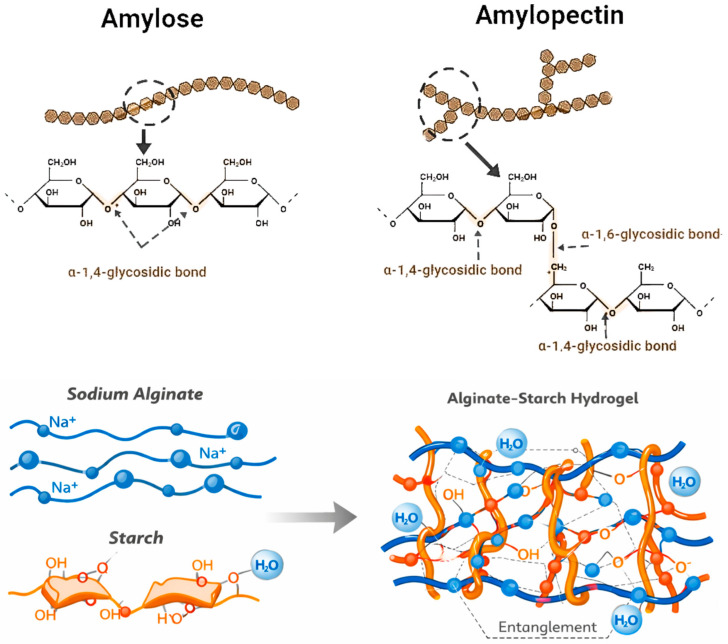
Structure of starch (amylose and amylopectin) as well as alginate–starch interactions.

Starch represents one of nature’s most abundant carbohydrates, essential for human nutrition and extensively utilized in various applications within the food industry [50]. It is chemically made up of two polymers: amylose, which has a linear structure, and amylopectin, which is branching [51,52]. The crystalline regions of starch granules are primarily composed of amylopectin, while amylose is predominantly located in the amorphous areas [51]. Amylose is a homopolymer composed of α-D-glucose units linked by α-1,4-glycosidic bonds, while amylopectin is a branched polymer composed of α-D-glucose units linked by α-1,4 and α-1,6 glycosidic bonds [51]. Figure 5 shows starch structure as well as alginate starch interaction with dominant hydrogen bonds and physical chain entanglements, while electrostatic and covalent bonds play a negligible role.

The ratio of starch components varies depending on their origin, influencing the physical and chemical properties of starch and allowing the formation of structures with specific characteristics [53]. Typically, starch consists of 20–30% amylose and about 70–80% amylopectin by weight [52]. The properties of starch are also influenced by other components contained in the starch granules, such as phosphates, lipids, and phospholipids [53].

According to its natural origin, starch can be classified into three main groups: cereal starches, tuber starches, and root starches [54]. Among cereal starches, corn starch has received particular attention in encapsulation and delivery systems due to its global availability, relatively low cost and well-characterized physicochemical properties. In addition, corn starch exists in several genetic and industrial varieties that differ considerably in amylose content, including waxy starch containing less than 5% amylose, normal starch with approximately 20–30% amylose, and high-amylose starch that may contain more than 50–70% amylose [55,56]. Such variability enables modulation of important functional properties, including swelling power, retrogradation tendency, gel firmness and diffusion characteristics, which are key parameters for designing encapsulation matrices and controlled release systems [56,57]. Furthermore, corn starch granules typically exhibit an intermediate size ranging from approximately 5 to 25 μm and predominantly A-type crystalline structure, which is characteristic of cereal starches and facilitates efficient gelatinization and formation of relatively compact and stable hydrogel networks when combined with other biopolymers such as alginate [55,56]. Due to these structural and functional characteristics, corn starch is frequently used as a representative cereal starch in studies investigating starch-based delivery systems. Another way to categorize starch is based on the type of crystalline lattice formed by amylopectin within the starch granules, which results in A-, B-, and C-type starches [54].

Because of its biocompatibility, non-toxicity, and biodegradability, starch is commonly used in hydrogel synthesis and as a matrix for encapsulating important bioactive compounds [58]. The presence of free hydroxyl groups in the starch structure allows for better water absorption, encapsulation efficiency, and regulated release of active compounds [58]. Besides encapsulation systems based on starch or its modified forms, combinations of starch with natural hydrocolloids, such as alginate, are increasingly being investigated [50]. The addition of starch in alginate carriers is of particular importance due to improving the encapsulation efficiency, reducing the permeability of the structure and enabling a more stable and controlled release of bioactive compounds [50,59]. The addition of starch to alginate-based hydrogels also significantly affects their mechanical, structural and rheological properties. Starch can affect the biopolymer network, tensile strength and water-holding capacity, while the type and properties of starch determine the final structure and stability of the gel. Gels composed solely of alginate exhibit higher stress, strain at break, and Young’s modulus values compared to alginate–starch gels, due to a higher concentration of alginate in the pure gel and the resulting greater number of carboxylate anions capable of binding calcium ions that act as cross-linking agents [59]. The addition of corn starch to alginate results in the formation of a dense, compact structure with small pores, because the amylose released during gelatinization forms hydrogen bonds with the carboxyl groups of alginate, thereby enhancing the overall strength of the gel [59]. Alginate gels enriched with potato starch exhibit lower mechanical strength, mainly due to the larger starch granules that create bigger pores within the gel network [59]. The increased granule size enhances water retention and swelling, which weakens the gel structure and reduces the number of junction zones, thereby lowering overall system stability. In contrast, gels containing cereal starches show higher stiffness, as gelatinization promotes more efficient separation of amylose and amylopectin chains, enabling better molecular organization and formation of a more compact and stable network [59]. Chemically modified corn starch can further alter gel behavior. For example, phosphorylated corn starch strengthens interactions between amylose and amylopectin chains, producing moderately brittle but more viscous and mechanically stronger gels [59]. Due to its low amylose content, this starch also exhibits greater thermal stability, making it suitable for canned, frozen, or refrigerated food products. Additionally, increasing the concentration of phosphorylated starch in alginate systems enhances gel elasticity [59].

Feltre et al. [60] reported that the type of corn starch significantly affects the properties of alginate gels. Gels containing gelatinized high-amylose corn starch exhibited higher stress and strain at break and greater Young’s modulus compared to gels prepared with ordinary or high-amylopectin gelatinized starch. This behavior is attributed to the strong retrogradation tendency of amylose, which forms double-helical structures and enhances intermolecular interactions with alginate, resulting in stronger gel networks. Gelatinized high-amylose starch also increased tensile strength compared to non-gelatinized starch, although no significant differences were observed after thermal treatment. Alginate gels containing gelatinized starches showed lower water-holding capacity than pure alginate gels due to the reduced alginate content responsible for forming the CaCl_2_-induced three-dimensional network [60].

In vitro digestion studies indicated that gels prepared with gelatinized starches had a more deteriorated microstructure and higher enzyme sensitivity than those containing ungelatinized starches. High-amylose starch produced more stable matrices during digestion and led to a more compact structure after the intestinal phase. Consequently, starch type influences release behavior: high-amylopectin gelatinized starches favor rapid intestinal release, whereas ungelatinized high-amylose starches are more suitable for dietary fiber delivery and compounds targeted for release or absorption in the colon [60].

The addition of natural corn starch increased the encapsulation effectiveness and stability of calcium alginate hydrogels [61]. The starch granules filled holes in the matrix, reducing the loss of active chemicals during particle formation. The addition of starch also influenced the uniform distribution of yerba mate (*Ilex paraguariensis*) extract within the particles, as well as a reduction in surface erosion, resulting in a more regulated and slower release of polyphenols [61]. Both systems (with and without starch) permitted full recovery of polyphenols following passage through simulated stomach and intestinal environments, despite variations in the initial release rate. Starch enhanced the extract’s retention capacity and controlled the release of antioxidants, but it had no effect on the extract’s antioxidant activity [61]. Alginate–starch microspheres containing *Lactobacillus rhamnosus* demonstrated strong encapsulation effectiveness (93.80 ± 0.63) and good sphericity, with an average size of 221.10 µm, which is the median value among other alginate systems [61]. This size and shape are regarded favorably because spherical and softer capsules provide a better sensory experience in food and have a reduced surface area exposed to oxygen, reducing oxidation during storage [62].

Modified porous starch, which has evenly distributed pores on the surface and good pH sensitivity, enables controlled release of active compounds under various conditions by altering the alginate-to-starch ratio [63]. Enzyme-modified porous starch has a prominent porosity structure and internal cavities that improve probiotic particle adherence while also filling the alginate gel’s interior space and allowing bacteria retention [63]. These features make a major contribution to improved encapsulation efficiency and thermal stability of the system [63].

Harper concluded that alginate films containing commercial modified potato starch exhibited significantly higher values of elongation at break, alongside lower puncture force, distance, and work values than control alginate films [64]. It is suggested that this commercial carbohydrate acted as a plasticizer in the film by disrupting alginate–alginate chain interactions [64]. Conversely, the addition of potato starch had no significant effect on the elongation of alginate films. Thus, while the addition of modified potato starch reduces the mechanical strength of the films, it simultaneously enhances their flexibility and extensibility [64]. Considering that certain sausage manufacturers utilize potato starch co-extruded alginate casings, these findings may hold practical implications for further potato starch application in this industry [64].

In addition to corn and potato starch, starches derived from other botanical sources such as rice, wheat, or tapioca have also been investigated as potential matrices for encapsulation and delivery systems. These starches differ considerably in granule morphology, amylose–amylopectin ratio, crystalline structure, and gelatinization behavior, which ultimately determine their functional performance in hydrogel networks [55,57]. For instance, rice starch is characterized by very small granules, typically ranging from approximately 2 to 8 μm, which provide a relatively large surface area and may facilitate faster hydration and diffusion of encapsulated compounds [55]. In contrast, potato starch granules are considerably larger, usually ranging from 15 to 100 μm, and exhibit a B-type crystalline structure, which is associated with a higher swelling capacity but often leads to weaker and more porous gel matrices [57]. Such structural differences among starch sources can significantly influence encapsulation efficiency, mechanical stability of hydrogels, and the release behavior of incorporated bioactive compounds [55].

Based on the literature presented, it can be concluded that the incorporation of starch, either native or modified, enhances the mechanical and rheological properties of hydrogels in a way that improves their suitability as carriers for the encapsulation of probiotics [62,63] and various bioactive compounds [50,61], as well as for ensuring their protection during gastrointestinal digestion [60,62].

### 2.4. Pectin

**Figure 6:**
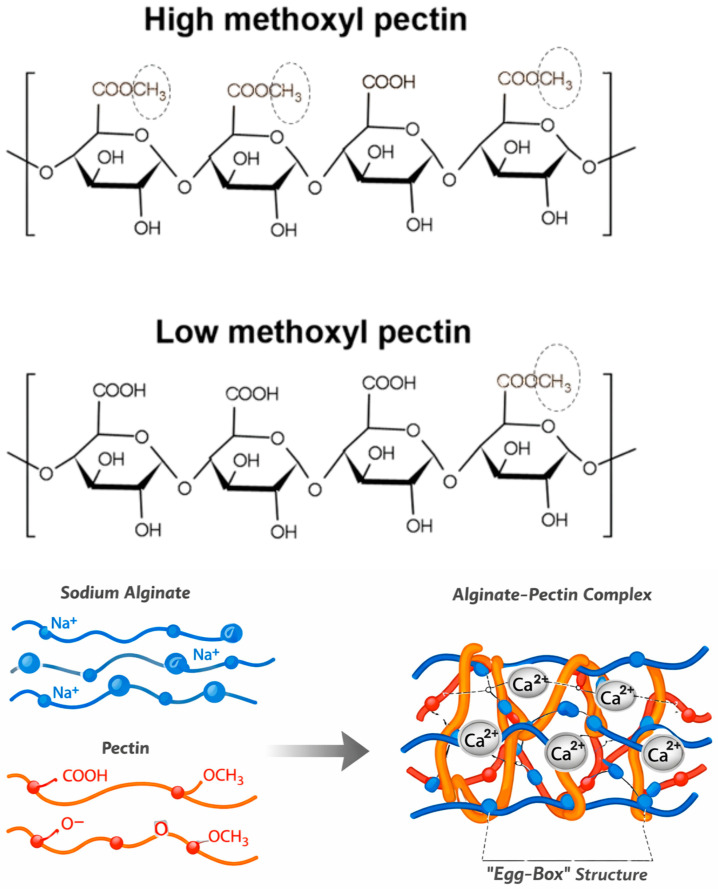
Structure of pectin (high methoxyl and low methoxyl) and interaction with alginate to create alginate–pectin hydrogel.

Pectin is an anionic heteropolysaccharide that is found in primary plant cell walls and the middle lamella [65]. Pectin’s chemical structure is made up of numerous polysaccharide associations, including homogalacturonans, xylogalacturonans, rhamnogalacturonans (types I and II), arabinans, and arabinogalactans [66]. The majority of its mass (60–70%) is made up of homogalacturonans, which are D-galacturonic acid polymers linked by α(1 → 4) glycosidic linkages [65,67]. Some carboxyl groups in the galacturonic acid molecule can be methyl-esterified, while others at the C-2 and C-3 positions are O-acetylated [66,67]. Citrus fruits (lemons, oranges, and limes) contain the most pectin in their pulp and peel, but sunflower, apple pomace, potatoes, tomatoes, carrots, and cocoa husks also have considerable amounts [65,68,69]. Commercial pectin is often derived from citrus peel and apple pomace as a byproduct of fruit juice production [65]. The structure of pectin is shown in Figure 6, as is the alginate–pectin interaction to create hydrogel with calcium ions. The dominant interactions between alginate and pectin are electrostatic (ionic) interactions between their carboxylate groups, supplemented by hydrogen bonding and polymer chain entanglement.

Pectin is easily extracted from natural sources using acid extraction protocols and water-based treatment [70,71]. Its chemical composition and properties vary depending on the plant source, and different forms of pectin have varied molecular weights, degrees of methylation and acetylation, and amounts of galacturonic acid and neutral sugar [68]. The esterification degree is a particularly essential feature influencing its physicochemical properties since it dictates pectin’s behavior throughout the gelation process as well as its rheological qualities [68,71]. Pectin with a high methyl-esterification degree readily forms gels in acidic environments at low pH [66,67,69]. In contrast, pectin with a low methyl-esterification degree is less sensitive to acidity and requires the presence of calcium ions for gel formation [68,71]. Pectin’s solubility is determined by various parameters, including methylation level, molecular weight, temperature, pH, and the presence of other ions in the solution [72]. Viscosity, gelling ability, and solubility are all related: circumstances that promote gel formation also increase viscosity while decreasing pectin solubility. This behavior results from pectin’s polycarboxylic structure [72]. Protonation of carboxyl groups happens at low pH, reducing ionization and hydration. As a result, it reduces the electrostatic repulsion between pectin strands, allowing them to approach, combine, and create a three-dimensional gel network [72]. The pK value of pectin depends on the esterification degree. Pectin with about 65% methyl-esterified groups has a pK of approximately 3.55, while completely unesterified pectin shows a pK of about 4.10, which indicates that pectin with a higher methylation degree gels at slightly higher pH, which is a consequence of a small number of free carboxyl groups available for ionization and the formation of ionic bonds [73].

Pectin’s chemical structure ensures great biocompatibility and bioavailability, making it ideal for a wide range of applications, from the food industry to biomedical systems [71]. Its capacity to form gels and robust network structures, as well as its strong synergistic interaction with other polysaccharides, particularly alginate, add to its value as a functional biopolymer [74,75]. Composite alginate–pectin hydrogels, formed by linking these polysaccharides, are particularly intriguing [73]. Their intermolecular interactions, which are based on unique chemical interactions between poly-G alginate blocks and methyl-esterified pectin segments, result in complexes that are more mechanically stable and biologically active. The stability and mechanical resilience of alginate–pectin gels are influenced by the spatial organization of junction zones, which form solid bands and double-layer crystalline structures [74]. The strongest gels are obtained when highly esterified pectin is combined with alginate rich in α-L-guluronic acid (G-blocks), suggesting interactions between methyl-esterified regions of polygalacturonic acid and alginate poly-G chains [74]. In addition to polymer composition, several environmental factors affect gel structure and properties, including polymer concentration, component ratio, sugar content, ion presence, and pH [74]. Maximum gel strength is typically achieved at an alginate–pectin ratio of approximately 1:1, while lowering the pH from 3.5 to 3.0 strengthens the gel network; further pH reduction has little additional effect. Higher pH values decrease the melting temperature, indicating that acidic conditions favor the formation of more stable gels [74]. Microstructural studies show that pure pectin gels exhibit a porous network with pores around 500 nm, whereas mixed alginate–pectin systems form more complex structures due to polymer chain aggregation during gelation [74]. These composite gels display higher surface roughness, a fibrous morphology with tightly packed fibers and small spherical particles (10–50 nm), and a quasi-square network structure, indicating strong polymer cross-linking and a synergistic interaction between alginate and pectin that contributes to high elasticity [74].

In vitro investigations have revealed that these gels progressively release bioactive compounds while maintaining their biological activity and stability [76]. Chen and Zhang [77] created biopolymeric gel microspheres with adjustable physical characteristics for the controlled release of proanthocyanidins. The resulting hybrid gel microsphere had high porosity and exceptional thermal stability, even at temperatures exceeding 140 °C, which was attributed to enhanced interactions via hydrogen bonds between molecular chains [77]. Samples with a higher proportion of pectin had more pronounced porosity, greater elasticity, better water solubility, and a higher moisture content in equilibrium conditions, whereas gels rich in alginate had more regular pores, increased mechanical resistance, and pronounced hydrophobicity [77]. The results of in vitro tests showed that proanthocyanidins incorporated in alginate–pectin gels were released gradually and in a controlled manner, with strong antioxidant activity. These microspheres showed a high ability to neutralize DPPH radicals and significant iron reduction power, with samples with a higher pectin content achieving the best results, confirming its role in improving the stability and biological efficiency of the encapsulated compounds [77]. The hydrogels based on pectin and alginate are effective carriers for probiotic protection and delivery during storage and passage through the gastrointestinal tract [29,78]. Pectin–alginate hydrogel supplemented with montmorillonite was used to form microspheres for *Lactobacillus kefiranofaciens* encapsulation [78]. The addition of montmorillonite contributed to the formation of a solid, mechanically stable cross-linked structure with a layered network on the surface, which enabled the stable encapsulation and protection of probiotic cells inside the microspheres while preserving their viability in the acidic environment of the stomach and the controlled gradual release of *L. kefiranofaciens* in the intestines, which resulted in efficient colonization of the large intestine [78]. Thanks to their porosity, structural stability and strong protective capacity, pectin–alginate hydrogels reinforced with montmorillonite represent highly promising materials for probiotic encapsulation in food products, especially in the dairy industry, where they can contribute to extending the shelf life and preserving the biological activity of beneficial microorganisms [78]. The differences in mechanical properties of an alginate–pectin carrier with and without flaxseed powder used for probiotic encapsulation is shown in Table 1. The alginate–pectin carrier exhibits the highest mechanical strength, whereas the alginate–flaxseed carrier demonstrates nearly two-fold lower strength, which is further reduced when alginate, pectin, and flaxseed are combined into a single matrix [29]. This indicates strong interactions between alginate and pectin that are disrupted by the addition of flax. However, the particles with the highest initial strength also exhibited the lowest stability during the fermentation process. The alginate–pectin carrier undergoes the most pronounced structural changes during fermentation and, despite being initially the strongest, becomes mechanically comparable to, or even weaker than, the alginate–flaxseed and alginate–pectin–flaxseed carriers [29]. These findings reflect the previously mentioned sensitivity of pectin to pH fluctuations occurring during fermentation, as well as the metabolic activity of the probiotics encapsulated within the matrix. In addition, the effectiveness of alginate and pectin hydrogels with different polymer ratios as carriers for folic acid was evaluated based on their release behavior in a simulated gastrointestinal environment [75]. The results showed that the hydrogel containing 70% alginate and 30% pectin was the most suitable in comparison with other combined hydrogels and pure alginate hydrogel [75]. The gel’s physical and chemical properties indicated the formation of a protective layer on the surface, while the spherical shape was preserved in samples with less than 60% pectin [75]. About 20% pectin sufficiently protected the matrix against the sudden release of folate in simulated gastrointestinal conditions, while a high pectin content reduced the mechanical strength and hindered the stability of the gel [75].

Based on the available research, it can be concluded that hydrogels produced from alginate–pectin combinations exhibit enhanced mechanical and rheological properties, making them a promising encapsulation system for probiotics, various oils, vitamins, tea extracts, and other bioactive compounds, as they provide protection during the manufacturing process and digestion, and enable controlled release [29,75,76,77,78].

### 2.5. Chitosan

**Figure 7:**
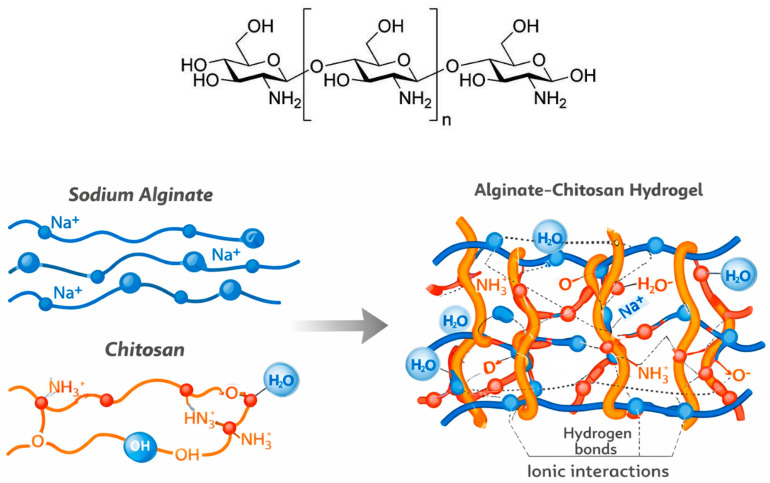
Chitosan structure and the alginate–chitosan interaction to create a composite hydrogel.

Chitosan is a polycationic polysaccharide derived from chitin, which is naturally present in the shells of crustaceans such as shrimp and crabs. Chemically, chitosan consists of amino polysaccharide chains produced through the deacetylation of chitin, which is, after cellulose, the second most abundant natural organic polymer [79]. Its polymeric structure is composed of D-glucosamine and N-acetyl-D-glucosamine units linked by β(1–4) glycosidic bonds [80,81]. The structure of chitosan is shown in Figure 7, as is the alginate–chitosan interaction to create a hydrogel. Chitosan is soluble in acidic aqueous solutions (pH < 6), enabling the formation of hydrogels. Its most beneficial characteristics are that it is biologically renewable, biodegradable, biocompatible, and non-toxic [82,83]. Chitosan can be easily enzymatically or chemically modified under physiological conditions and processed into a variety of forms. In addition, chitosan exhibits intrinsic antibacterial, fungistatic, and anti-inflammatory properties [83,84]. It is also hemostatic, and through ionic interactions with negatively charged red blood cells and platelets, it can rapidly induce blood clotting [83,84]. Due to these properties, chitosan has great potential for food, environmental, and pharmaceutical applications.

However, several challenges limit its translation from research to commercial products, including food applications. Due to its origin and potential allergenicity (e.g., shellfish allergies), regulatory approval for chitosan as a food additive is not harmonized globally. Furthermore, significant batch-to-batch variability affects its average molecular weight, which in turn alters its bioactive properties and processability [85]. Finally, the production of high-purity, food-grade chitosan with controlled molecular weight is costly due to the complex extraction and purification steps.

Since chitosan is positively charged, it readily forms a polyelectrolyte complex with alginate, an anionic polysaccharide, resulting in synergistic chemical, rheological, and mechanical behavior [86]. Their interaction is driven primarily by proton transfer and electrostatic attraction between protonated amine groups of chitosan and carboxylate groups on alginate. The pK_a_ of chitosan’s amino groups is approximately 6.5, whereas the pK_a_ of alginate’s carboxyl groups is approximately 3.5, indicating that these complementary ionization profiles enable the formation of composite hydrogel with a broader pH stability range (approximately pH 3–7) compared to either polymer alone [86].

There are two mechanisms to create alginate–chitosan gels [87]: coating of alginate gel by chitosan, and blending of chitosan and alginate during the gelation process. Chitosan coatings produce dense polyelectrolyte membranes that reduce permeability and enhance stability, whereas blending tends to produce particles with higher mechanical resistance. High-molecular-weight chitosan (415 kDa) produces gels with higher mechanical stability compared to those produced using medium-molecular-weight chitosan (103 kDa) [88]. In addition, gel stiffness increases as pH decreases from 7 to 5 due to enhanced protonation of chitosan and stronger electrostatic attraction with deprotonated alginate [89]. As a result, the network becomes more rigid, with increased Young’s modulus and overall stiffness at higher chitosan concentrations [90].

Hydrogels based on chitosan–alginate polyelectrolyte complexes, owing to their biocompatibility, biodegradability, and non-toxicity, have been extensively investigated for the encapsulation of various bioactive compounds, including vitamins, proteins, aromas, drugs, and living cells, with promising results [91,92]. These composites effectively protect the encapsulated agents and are therefore considered efficient delivery systems [93]. Vitamins and antioxidants such as omega-3-rich oils are essential nutrients but are highly sensitive to adverse environmental factors (e.g., temperature, oxygen, light, and moisture), making them prone to substantial degradation during food processing and storage. Encapsulation into micro- or nano-sized spherical particles is one of the most effective strategies to preserve their stability by minimizing oxidation and degradation. For instance, alginate–chitosan nanoparticles have been shown to efficiently retain vitamin B2, remaining stable for at least five months [94]. This stability is attributed to interactions between vitamin B2 and the biopolymers: in addition to ionic interactions with alginate, vitamin B2 can engage in Van der Waals forces and hydrogen bonding with both alginate and chitosan [94,95]. Furthermore, alginate–chitosan hydrogels have proven suitable as edible carriers for hydrophobic nutraceuticals such as omega-3-rich oils (e.g., flaxseed or fish oil), with the aim of reducing oxidation and maximizing their health benefits [96]. In addition, alginate–chitosan matrices are widely used for the encapsulation of essential oils and aromas, addressing challenges such as volatility, instability, and poor water solubility [97,98,99]. The intrinsic antimicrobial activity provided by chitosan enables these composite hydrogels to function as edible coatings [100,101,102] or food-packaging films [103,104,105], helping to reduce spoilage and extend freshness. In probiotic encapsulation, chitosan is typically used for coating particles that already contain encapsulated probiotics, rather than as a component of the carrier formulation itself, primarily due to its bacteriostatic properties. Krunic et al. [4] demonstrated that coating an alginate carrier with chitosan significantly decreases hydrogel porosity and increases mechanical strength and stability during fermentation (Table 1). Table 1 shows that the addition of chitosan has the greatest impact on the mechanical strength, stability and porosity of the alginate carrier compared to whey proteins/peptides and pectin.

## 3. Conclusions

Although alginate hydrogels are widely used due to their mild ionic gelation and food-grade status, their intrinsic limitations, such as high porosity, limited mechanical stability, and weak resistance to acidic environments, often restrict their performance as delivery systems. The reviewed literature demonstrates that alginate-based hydrogels, when combined with complementary biopolymers, provide an effective strategy for tailoring hydrogel functionality, thus offering significant advantages for the encapsulation and delivery of probiotics and a wide range of bioactive compounds. Table 2 summarizes the main impact of biopolymer addition in alginate described in the paper.

Among these systems, alginate–whey protein matrices exhibit pH- and micelle-dependent interactions that enhance gel network density, encapsulation efficiency, and protection of sensitive bioactives and probiotics, but their performance may vary depending on protein fraction and processing conditions. Alginate–gelatin blends, in contrast, form more predictable and stable networks through electrostatic and hydrogen-bond interactions, providing controlled release and robust structural integrity suitable for edible films, coatings, and emerging 3D-printing applications.

Polysaccharide-based combinations offer complementary advantages: starch reinforces mechanical and rheological performance, while pectin enhances hydrogel stability and protective capacity for oils, vitamins, and polyphenols. Alginate–chitosan complexes contribute additional functionalities, including antimicrobial activity and improved structural integrity, particularly valuable in active packaging and probiotic encapsulation.

Overall, this review highlights that the functional outcomes of alginate-based hydrogels depend critically on the type of biopolymer incorporated, the nature of the polymer–polymer interactions, and the target application. Rather than providing a one-size-fits-all solution, the integration of alginate with proteins, polysaccharides, or chitosan represents a toolbox for designing multifunctional composite hydrogels with tunable mechanical strength, stability, and controlled-release behavior, offering a versatile platform for the development of functional foods and nutraceutical products.

## Figures and Tables

**Table 2:** Comparison of polymer additives in alginate-based composite hydrogels.

Polymer Additive	Main Interactions with Alginate	Microstructure Effects	Functional Outcome	Limitations	References
Whey protein (WPI/WPC/WPH)	Electrostatic interactions; hydrogen bonding; Ca^2+^ bridging; hydrophobic interactions	Denser gel network; improved emulsion stabilization	↑ mechanical strength; ↑ encapsulation efficiency; ↑ stability of lipophilic compounds	Sensitivity to pH and heat; possible structural heterogeneity	[19,21,22,23,24,25,28]
Gelatin	Hydrogen bonding; electrostatic interactions; thermoreversible gelation	Flexible network; possible phase separation depending on pH and polymer ratio	↑ elasticity; controlled release; improved bead integrity	Temperature sensitive; limited acid resistance	[33,36,37,38,41,47,48]
Starch (native/modified)	Hydrogen bonding; pore filling; amylose retrogradation	Reduced pore size; compact structure	↑ encapsulation efficiency; ↑ digestion resistance; tunable swelling	Gel weakening with high amylopectin content; sensitive gelatinization conditions	[50,55,57,59,60,61,62,63,64]
Pectin (LM/HM)	Hydrogen bonding; Ca^2+^ bridging (LM pectin); network synergy	Fibrous network; enhanced gel stability	↑ stability in acidic conditions; controlled release; ↑ antioxidant retention	Excess pectin may reduce mechanical strength	[29,73,74,75,76,77]
Chitosan	Strong electrostatic complexation	Dense coating or reinforced matrix	↓ porosity and permeability; ↑ mechanical stability; antimicrobial properties	Possible reduction in probiotic viability at high concentration	[4,85,86,87,88,89,90,91,92,93,100,101,102]

WPI—whey protein isolate; WPC—whey protein concentrate; WPH—whey protein hydrolysate; LM—low methoxyl pectin; HM—high methoxyl pectin, ↑—increase, ↓—decrease.

## Data Availability

No new data were created or analyzed in this study.
